# Analysis of elite soccer players’ performance before and after signing a new contract

**DOI:** 10.1371/journal.pone.0211058

**Published:** 2019-01-25

**Authors:** Miguel-Ángel Gómez, Carlos Lago, María-Teresa Gómez, Philip Furley

**Affiliations:** 1 Faculty of Physical Activity and Sports Sciences, Technical University of Madrid, Madrid, Spain; 2 Faculty of Education and Sport Sciences, University of Vigo, Pontevedra, Spain; 3 Institute of Cognitive and Team/Racket Sport Research, German Sport University Cologne, Cologne, Germany; Universidade de Tras-os-Montes e Alto Douro, PORTUGAL

## Abstract

The aim of the current study was to analyse performance differences of football players 2-years prior and the year after signing a new contract (the following year) while taking playing position, nationality, player’s role, team ability, and age into account. The sample was comprised of 249 players (n = 109 defenders, n = 113 midfielders; and n = 27 forwards) from four of the major European Leagues (Bundesliga, English FA Premier League, Ligue 1, and La Liga) during the seasons 2008 to 2015. The dependent variables studied were: shooting accuracy, defense (the sum of defensive actions, tackles, blocks, and interceptions), yellow cards, red cards, passing accuracy, tackle success, and minutes played per match. Two-step cluster analysis allowed classifying the sample into three groups of defenders (national important, foreign important, and less important players) and four groups of midfielders and forwards (national important, foreign important, national less important, and foreign less important players). Magnitude Based Inference (MBI) was used to test the differences between player’s performances during the years of analyses. The main results (very likely and most likely effects) showed better performance in the year prior to signing a new contract than the previous year for foreign important defenders (decreased number of red cards), national important midfielders (increased number of minutes played), foreign important forwards (increased minutes played and defense), and national important forwards (increased minutes played). In addition, performance was lower the year after signing the contract compared to the previous one for less important defenders (decreasing defense), national less important midfielders (decreased minutes played), and foreign less important forwards (decreased defense). On the other hand, the players showed better performance in defense and more minutes played the year after signing the contract for less important defenders, national less important midfielders, and foreign less important forwards. These results may assist coaches to decide on when a new contract should be signed or the duration of the contract.

## Introduction

Incentives are known to play a substantial role in a person’s performance. An important incentive in professional football is signing a lucrative contract. While performance analysis in football has focused on numerous variables in explaining performance, there is limited knowledge on the impact of the time of signing a contract in football. Previous research on performance analysis in football has investigated the players’ performance from an individual point of view attending to physical demands [1] and technical and tactical indicators [2] according to different performance levels [3], playing positions [4–5], players’ role as starters or non-starters [6], the evolution of physical and technical parameters [7–8] or performance variability [9]. However, a limitation of this previous research has been to neglect a long-term analysis of a player’s individual performance [10]. Of further relevance to the present research, Mackenzie and Cushion [11] have argued that research on performance analysis should identify long term constraints on an individual’s performance which can be used to improve a player’s recruitment policies and to control for social-cultural (e.g., foreign/ national, top elite leagues) influences that impact players’ performance during their careers.

Pertinent to the present research, football players have different fixed contracts that have been argued to affect their efforts with cycles of performance variation at different moments of their career [12]. Research within sport management has shown that this is particularly evident just before a player signed a new contract (i.e., better performance) and after a lucrative contract was secured (i.e., maintenance or reduction of performance) [12–13]. Frick [12] studied 1,993 players from the German Bundesliga during the seasons 1995–1996 to 2007–2008 (13 seasons). The results showed that career matches played, matches played during the last season of contract, goals scored, yellow cards, playing position, and region of birth varied depending on signing a new contract as this seemed to have affected the player’s effort and motivation. Della Torre et al. [13] analysed 275 football players who played at least two consecutive seasons (year before and after signing a contract) in the Italian Serie A during the seasons 2012–2013 and 2013–2014. Their results showed that players perform better during the last year of their contract which is argued to be caused by top elite teams rewarding current/past performances (i.e., subsequent contracts are specially affected by the immediate past performance). Their results showed a pay-performance (current salary) relationship that reinforces good performances. However, this factor has disparities when controlling for player’s origin (national or foreign player) and their level of performance based on performance indicators (important or less important players). In fact, their results showed that domestic players performed better than foreign players due to the better knowledge of the culture of the country, the league, and the club. Accordingly, as football players sign new contracts of about three years of duration or renegotiate their contract with one or two years remaining, the performance analysis of players using key performance indicators is of great relevance to understand the cycle of efforts and motivation based on current contracts, renegotiation, and salary [13].

Of further relevance to the present research, player-related factors obviously impact on players’ performance. For example, the nationality of players has been argued to be a moderator on the performance effects of signing a new contract in elite football and has been studied from social and management perspectives [14]. More recently, Della Torre et al. [13] found that the individual performance during consecutive seasons was stronger for domestic players than foreign players when the end of the contract is near. In addition, a player’s age, his role in the team (categorized based on minutes played as important or less important), the strength of the team in which they play (UEFA ranking) or the evolution of their technical-tactical performances (such as passing, shooting, tackling, or defending behaviours) during the previous and posterior performances after signing or renegotiating a contract may affect the player’s performance from a long-term approach [2–3, 6, 8–9, 12–14].

In summary, research has indicated that a player is willing to invest more effort when approaching the renegotiation of the same or a new contract. Data shows that this shows in a consecutive increase of his performances mainly during the previous year(s) of his contract [12]. Presumably this gradual process to deliver better performance allows reaching a better bargaining position for the new contract during the last season before his contract expires or to renegotiate the current contract with one or two years remaining.

In the present paper, we investigate player’s performance variations using technical and tactical performance indicators that arguably reflect the evolution of his efforts in different areas of playing (defense, attack or minutes played). Further, due to the limit of 2-year analyses of previous studies, we will analyse players’ efforts during three consecutive seasons as a novel approach to gaining a better understanding of the signing/ renegotiating of a contract from a longer-term perspective. This analysis will allow illuminating players’ tendencies of adjusting their efforts depending on the remaining years of their contract [12]. These analyses are likely to be of interest for stakeholders (i.e., coaches, players, managers, and media) in elite football [13]. Therefore, the current study tries to address the limitations of previous studies (i.e. only using 2-years period of analyses, not using minutes played by the players and key performance indicators, or the omission of some player-related factors that have the potential to affect long-term performance). Thus, the aim of the current study was to analyze differences in performances of individual football players according to the previous (2-years) and the later year after signing/ renegotiating a new contract while taking player-related characteristics into account (age, role in the team as important or less important, nationality, and team’s ability). We hypothesized that performance during the previous season is better (as indicated by the following performance indicators: shooting accuracy, defense, yellow cards, red cards, passing accuracy, tackle success, and minutes played per match) than performance immediately after signing/ renegotiating the new contract. Additionally, we assumed that this should be more pronounced in domestic than foreign players.

## Materials and methods

### Subjects

The sample was comprised of 249 players (n = 109 defenders, n = 113 midfielders; and n = 27 forwards) from the French, German, Italian and Spanish professional leagues during the seasons 2008 to 2015. The distribution of players and total match observations for each league and playing position are presented in Table 1. The use of the sample from 4 of the major leagues of Europe allows increasing the number of observations of Elite athletes that compete at the same level of performance (European professional leagues). This approach follows from previous research, while further reducing the omitted variables bias (less statistical risks) when increasing sample sizes of the same performance characteristics [15–17].

**Table 1:** Number of players studied according to league and playing position (n and total matches played).

	Defender		Midfielder		Forward	
League	Players	Matches	Players	Matches	Players	Matches
Germany	24	1,730	24	1,780	8	522
Spain	26	2,017	26	2,120	4	334
France	29	2,097	29	2,378	8	652
England	30	2,286	34	2,573	7	568
Total	109	8,130	113	8,851	27	2,076

The players were selected if they played at least 20 matches (with more than 20 minutes per match) per season and completed a consecutive 3-years period that includes the year two seasons before the end of their contract (year -1), the last year of the contract (year 0) and the year immediately after signing a new contract (year 1). The year when players signed the new contract (considering either when it was renegotiated or when moving to another club) was considered as the reference year (year 0) in order to establish 2 years prior to sign (year -1), and the year after signing the contract (year 1). This allows to compare potential performance improvement due to the new contract with previous years.

The data included the mean performance of 249 players during three seasons (n = 747 mean individual observations). The following player-related characteristics that may affect performance depending on playing position (established by the official webpage of Opta Sports Company considering: defender, midfielder and forward) were: (i) age (year old of player during the year 0), (ii) team ability (UEFA ranking of the team where the player play during the year 0), (iii) player nationality (classified as national or domestic players of their respective leagues), and (iv) player’s role (classified by a *k*-means cluster using minutes played per match during the year 0 as important: 80.32±6.2 minutes; and less important players: 52.55±12.0 minutes).

### Design

The data observations were provided by OPTA Sportsdata Spain Company (S1 Dataset). The tracking system of this private company was previously tested by Liu, Hopkins, Gómez and Molinuevo [18] with acceptable inter-operator reliability. The study does not present the name of the players in order to keep the anonymity following the Company Ethics guidelines, the European Data Protection Law and the approval of the Institutional Review Board (Technical University of Madrid).

The variables (7 variables) were selected because they are arguably the most used performance indicators in the previous literature [2,18]. They have an established impact on individual performance and they can be used numerically to compare footballers over consecutive seasons [2,18]. The seven variables were defined as follows (https://www.optasports.com/news/optas-event-definitions/) [18]:

- *Shooting accuracy* (%): shots on target divided by all shots (including blocked attempts).
- *Defense*: the sum of defensive actions including tackles (“where a player connects with the ball in a ground challenge where he successfully takes the ball away from the player in possession”), blocks (This variable includes *blocked passes*: “when a player tries to cut out an opposition pass by any means. Similar to an interception except there is much less reading of the pass”; and *blocks*: “where a player blocks a shot on target from an opposing player”), and interceptions (“where a player reads an opponent’s pass and intercepts the ball by moving into the line of the intended pass”).
- *Yellow cards*: The yellow cards booked by the referee due to rule violations.
- *Red cards*: the red cards booked by the referee due to rule violations.
- *Passing accuracy* (%): successful passes divided by total attempted passes (considering all the types and zones of passes, excluding crosses).
- *Tackles success* (%): successful tackles divided by total attempted tackles.
- *Minutes per match*: the number of minutes played during a season divided by the number of matches played during the season.

### Statistical analysis

First, the *k*-means cluster for quantitative variables was used in order to establish cut-off point values for the variable minutes played per match. Then, two cluster were identified for this variable establishing important (n = 188) and less important (n = 61) players. Specifically, this model allows to divide *n* players’ observations into *k* clusters (groups) where each observation gets allocated to the cluster with the closest mean value.

Second, a two-step cluster analysis was used to classify the players into different categories based on player-related characteristics (age, team ability, player’s nationality and player’s role). The model allows the inclusion of categorical and continuous variables in order to find the best clustering solution. Then, this statistical analysis automatically determines the "optimal" number of clusters (player’s groups) using the Schwartz’s Bayesian Information criterion (Silhouette measure of clusters cohesion and separation and the variables importance). In addition, the log-likelihood distance measure was used to compute the similarity between clusters. Due to the non-significant effect when classifying the groups depending on team’s ability (variable importance = 0.0), the model was run without this variable (Silhouette measure indicated good results of 0.75, 0.70 and 0.68 for defenders, midfielders and forwards, respectively). Then, the sample was split into three groups of defenders (national important, foreign important, and less important players) and four groups for midfielders and forwards (national important, foreign important, national less important and foreign less important players). Table 2 shows the results (distribution of players) of this two-step cluster analysis for all the playing positions.

**Table 2:** Results of the clusters identified by the two-step cluster analysis for all the playing positions (I = importance of variables when classifying the players).

	**Cluster 1** (National important)	**Cluster 2** (Less important)	**Cluster 3** (Foreign important)	
**Defenders**				
N (%)	52 (47.7%)	7 (6.4%)	50 (45.9%)	
Player’s role (I = 1.0)	Important (100%)	Less important (100%)	Important (100%)	
Nationality (I = 0.94)	National (100%)	National (57.1%)	Foreign (100%)	
Age (I = 0.2)	31.29±3.9	32.71±2.3	32.26±3.7	
	**Cluster 1** (National Less important)	**Cluster 2** (National important)	**Cluster 3** (Foreign important)	**Cluster 4** (Foreign Less important)
**Midfielders**				
N (%)	26 (23%)	43 (38.1%)	30 (26.5%)	14 (12.4%)
Nationality (I = 1.0)	National (100%)	National (100%)	Foreign (100%)	Foreign (100%)
Player’s role (I = 1.0)	Less important (100%)	Important (100%)	Important (100%)	Less important (100%)
Age (I = 0.1)	32.12±3.8	30.65±4.7	31.40±3.4	31.07±2.9
	**Cluster 1** (Foreign important)	**Cluster 2** (Foreign Less important)	**Cluster 3** (National important)	**Cluster 4** (National Less important)
**Forwards**				
N (%)	7 (25.9%)	8 (29.7%)	6 (22.2%)	6 (22.2%)
Nationality (I = 1.0)	Foreign (100%)	Foreign (100%)	National (100%)	National (100%)
Player’s role (I = 1.0)	Important (100%)	Less important (100%)	Important (100%)	Less important (100%)
Age (I = 0.1)	32.0±4.2	31.25±4.1	30.83±3.0	30.83±4.7

Third, these groups were considered as the independent variable when comparing the performance indicators (dependent variable). Then, the player’s performance during the years (-1, 0 and 1) was analyzed using repeated measures ANOVA for normally distributed variables and the Friedman test for non-normally distributed variables. In addition, the pairwise comparisons (years -1, 0 and 1) were tested using the magnitude-based inference (MBI) method with the Hopkins’ spreadsheet [19–20]. This method uses the log-transformation of data in order to reduce bias due to non-uniformity error. The effect size (Cohen's *d* units at 90% CI) was estimated using pooled standard deviation for comparisons with the following magnitude ranges: 0–0.2 trivial; >0.2–0.6 small; >0.6–1.2 moderate; >1.2–2 large; >2 very large. The MBI analyses were assessed using the smallest worthwhile difference (0.2 times the standardization), estimated from the between-subjects standard deviation. The differences are defined as unclear if the confidence intervals (CI) for the difference in the means included substantial positive and negative values (±0.2*standardization) simultaneously. In order to control for differences between pairs of comparisons (years), the magnitude of a clear difference was assessed as follows: >0.25 trivial; 0.25%–75% possibly, 75%–95% likely, 95%- 99% very likely, and >99% most likely. The magnitude is considered unclear if the CI overlaps the positive and negative thresholds [19–20].

The descriptive results, repeated measures ANOVA, Friedman test and Two-step cluster analyses were performed using the statistical software IBM SPSS for Windows, version 22.0 (Armonk, NY: IBM Corp.).

## Results

The descriptive results (means and standard deviations), the repeated measures ANOVA, and Friedman tests results of the variables studied for defenders according to the player’s characteristics during the years prior to the end of their contract and immediately after signing a new contract are presented in Table 3. The repeated measures tests showed significant differences (p<0.05) of red cards for important national defenders, yellow cards and minutes played for less important defenders, and defense for foreign important defenders.

**Table 3:** Descriptive results (mean and standard deviations) for defender players.

DEFENDERS	Year -1		Year 0		Year 1		P
	M	SD	M	SD	M	SD	
National important							
Shooting accuracy †	0.32	0.25	0.40	0.20	0.35	0.22	0.14
Defence †	154.73	86.67	163.81	78.73	176.08	89.67	0.64
Yellow cards †	4.40	3.31	5.25	3.55	5.22	3.52	0.54
Red cards †	0.40	0.69	0.31	0.51	0.16	0.37	0.07
Passing accuracy *	0.75	0.08	0.76	0.09	0.75	0.08	0.71
Tackle accuracy *	0.78	0.08	0.76	0.07	0.79	0.07	0.09
Minutes per match †	78.32	10.80	81.80	5.78	82.11	9.68	0.06
Less important							
Shooting accuracy †	0.31	0.17	0.29	0.16	0.49	0.24	0.19
Defence †	102.14	51.10	74.86	36.15	120.86	69.26	0.06
Yellow cards †	2.71	1.98	2.83	3.54	4.14	3.80	0.03
Red cards †	0.29	0.49	0.33	0.52	0.43	0.79	0.66
Passing accuracy *	0.75	0.08	0.75	0.09	0.75	0.08	0.78
Tackle accuracy *	0.77	0.13	0.80	0.08	0.82	0.11	0.68
Minutes per match †	73.20	11.60	60.10	2.50	73.60	8.17	0.01
Foreign Important							
Shooting accuracy †	0.31	0.20	0.36	0.27	0.38	0.19	0.13
Defence †	178.80	104.87	187.88	85.52	228.88	104.00	0.01
Yellow cards †	3.27	2.12	3.80	2.88	4.29	2.71	0.12
Red cards †	0.18	0.44	0.50	0.66	0.29	0.54	0.08
Passing accuracy *	0.77	0.07	0.78	0.06	0.78	0.06	0.48
Tackle accuracy *	0.78	0.07	0.78	0.07	0.79	0.07	0.89
Minutes per match †	80.77	8.22	82.18	6.01	82.75	11.84	0.13

* p-values of repeated measures ANOVA;

^†^ p-values of Friedman non-parametric test.

**Fig 1:**
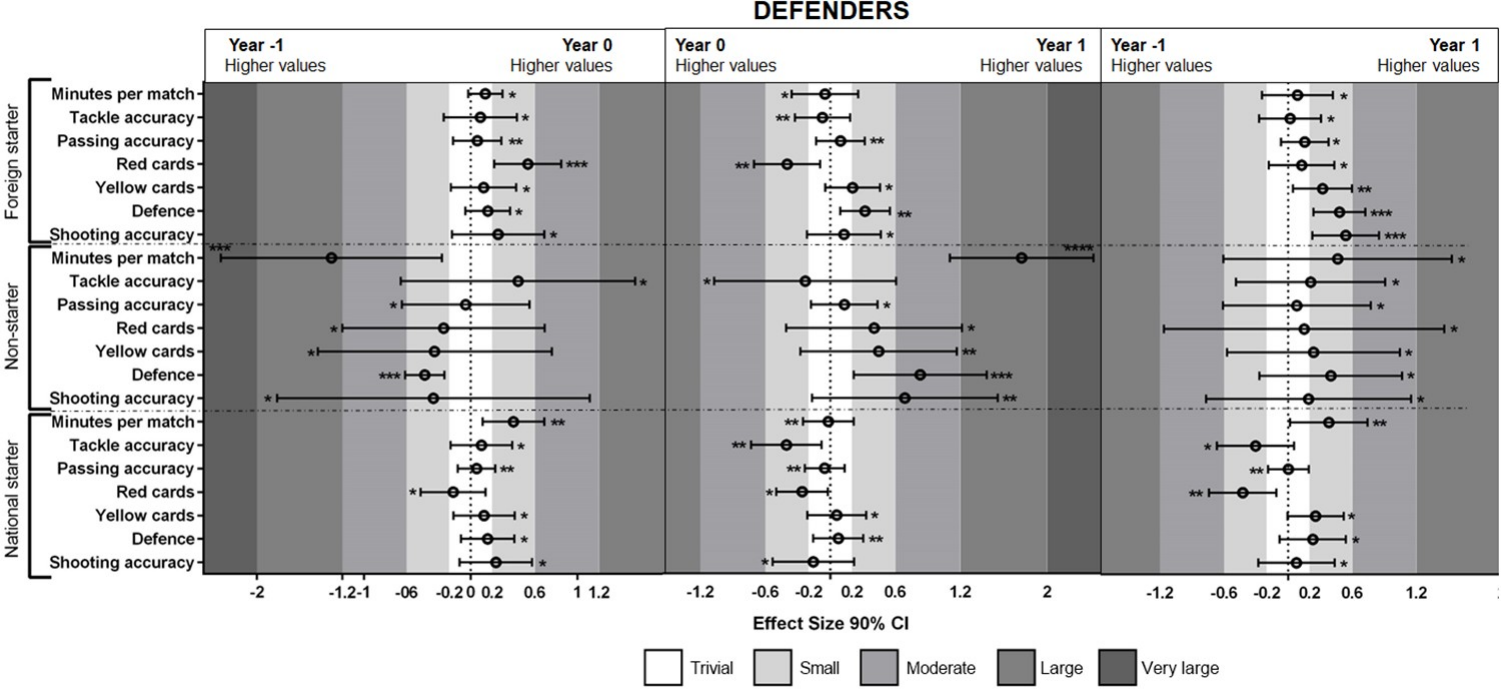
Standardized (Cohen’s d) differences between defender players’ performances during year 0 vs. year -1, year 1 vs. year 0 and year 1 vs. year -1. Asterisks indicate the likelihood of MBI effects as follows: *possibly, **likely, ***very likely, **** most likely.

The results of MBI (see Fig 1) showed that foreign important players decreased the number of red cards (very likely effect) from year -1 to year 0. The results for less important players showed a decreased performance in defense (very likely effect) from year -1 to year 0 and they increased their performance in the number of minutes played (most likely effect) and in defense (very likely effect) from year 0 to year 1.

The descriptive results (means and standard deviations), the repeated measures ANOVA, and Friedman tests results for midfielder according to the group of players during the years studied are presented in Table 4. The results showed statistically significant differences (p<0.05) of defense and minutes played for national less important players and minutes played for national important midfielders.

**Table 4:** Descriptive results (mean and standard deviations) for midfielder players.

MIDFIELDERS	Year -1		Year 0		Year 1		
	M	SD	M	SD	M	SD	P†
National less important							
Shooting accuracy	0.41	0.15	0.40	0.19	0.39	0.15	0.29
Defence	55.19	46.26	35.04	20.90	69.85	46.34	0.01
Yellow cards	3.62	2.91	3.48	3.04	4.28	3.18	0.33
Red cards	0.12	0.33	0.08	0.28	0.16	0.37	0.32
Passing accuracy	0.73	0.09	0.73	0.09	0.73	0.08	0.76
Tackle accuracy	0.78	0.09	0.74	0.09	0.75	0.08	0.34
Minutes per match	64.33	15.76	51.36	13.48	69.01	16.09	0.01
National important							
Shooting accuracy	0.41	0.19	0.38	0.16	0.43	0.15	0.14
Defence	82.02	50.45	88.91	49.05	89.72	61.27	0.61
Yellow cards	4.57	2.34	5.60	3.62	5.08	2.39	0.21
Red cards	0.19	0.45	0.19	0.45	0.26	0.55	0.60
Passing accuracy	0.77	0.08	0.76	0.08	0.77	0.08	0.12
Tackle accuracy	0.75	0.07	0.77	0.05	0.77	0.05	0.85
Minutes per match	71.68	12.39	78.67	6.21	74.39	12.37	0.04
Foreign less important							
Shooting accuracy	0.44	0.19	0.39	0.16	0.45	0.18	0.58
Defence	60.50	44.22	82.40	65.84	79.17	45.31	0.60
Yellow cards	4.40	3.23	4.54	2.19	4.36	2.68	0.88
Red cards	0.27	0.52	0.11	0.31	0.32	0.61	0.19
Passing accuracy	0.76	0.07	0.76	0.07	0.76	0.06	0.67
Tackle accuracy	0.77	0.08	0.78	0.07	0.79	0.07	0.11
Minutes per match	68.36	15.36	78.35	5.48	73.27	13.27	0.07
Foreign important							
Shooting accuracy	0.42	0.16	0.41	0.19	0.47	0.13	0.36
Defence	56.29	53.11	38.71	26.72	43.71	33.07	0.57
Yellow cards	2.85	2.30	3.23	2.77	2.92	1.62	0.89
Red cards	0.31	0.63	0.15	0.38	0.08	0.29	0.99
Passing accuracy	0.77	0.08	0.75	0.09	0.76	0.08	0.61
Tackle accuracy	0.78	0.06	0.80	0.07	0.82	0.11	0.26
Minutes per match	65.93	13.78	55.46	9.06	60.91	18.35	0.14

^†^ p-values of Friedman non-parametric test.

**Fig 2:**
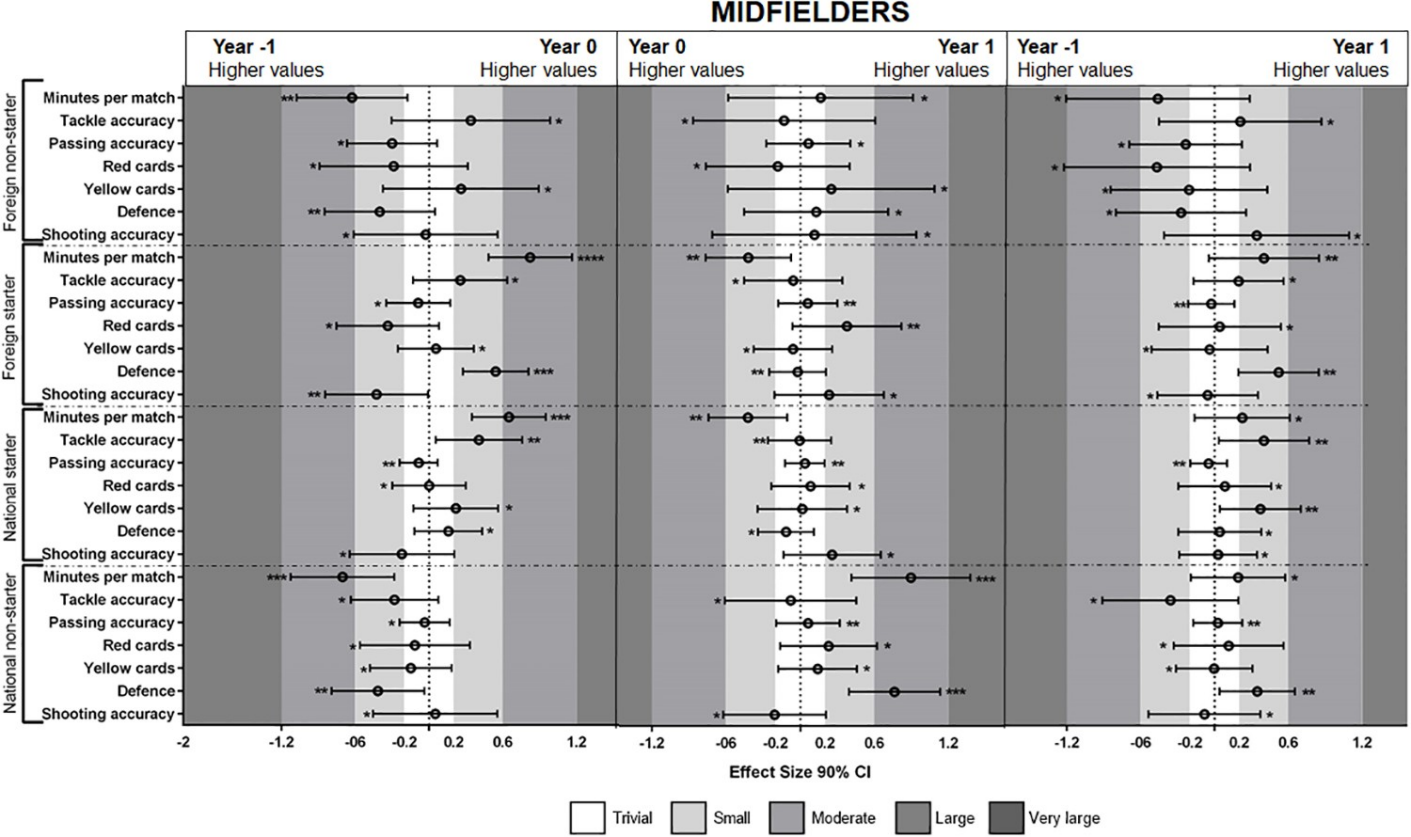
Standardized (Cohen’s d) differences between midfielders players’ performances during year 0 vs. year -1, year 1 vs. year 0 and year 1 vs. year -1. Asterisks indicate the likelihood of MBI effects as follows: *possibly, **likely, ***very likely, **** most likely.

The MBI results for midfielders (see Fig 2) showed that national less important players decreased the minutes played (very likely effect) from year -1 to year 0. Additionally, the players increased the minutes played (very likely effect) and defense from year 0 to year 1 (very likely effect). The results for national important players showed an increase in the number of minutes played from year -1 to year 0 (very likely effect). The foreign important players increased the minutes played and defense performance (very likely effect) from year -1 to year 0.

The descriptive results (means and standard deviations) and the Friedman tests results for forwards according to the group of players during the years studied are presented in Table 5. Statistical significant results (p<0.05) were identified in minutes played for foreign and national important players, for passing accuracy in national less important forwards, and in defense performance for foreign less important forwards.

**Table 5:** Descriptive results (mean and standard deviations) for forward players.

FORWARDS	Year -1		Year 0		Year 1		
	M	SD	M	SD	M	SD	P
Foreign important							
Shooting accuracy †	0.52	0.07	0.49	0.09	0.42	0.19	0.10
Defence †	28.86	27.03	24.29	7.25	23.86	16.42	0.86
Yellow cards †	1.67	1.21	3.71	2.29	1.33	1.21	0.06
Red cards †	0.00	0.00	0.14	0.38	0.00	0.00	0.99
Passing accuracy *	0.72	0.05	0.70	0.06	0.70	0.06	0.66
Tackle accuracy †	0.69	0.32	0.76	0.15	0.81	0.17	0.65
Minutes per match †	63.06	16.54	75.68	7.53	47.47	22.31	0.02
Foreign less important							
Shooting accuracy †	0.51	0.12	0.47	0.06	0.49	0.08	0.25
Defence †	25.63	12.60	14.00	7.37	36.63	24.63	0.03
Yellow cards †	2.88	2.17	2.14	1.95	3.57	2.23	0.06
Red cards †	0.38	0.52	0.00	0.00	0.14	0.38	0.32
Passing accuracy *	0.69	0.06	0.68	0.07	0.67	0.09	0.26
Tackle accuracy †	0.75	0.15	0.85	0.11	0.82	0.09	0.53
Minutes per match †	61.41	24.46	45.56	13.92	68.75	13.69	0.09
National important							
Shooting accuracy †	0.49	0.10	0.49	0.05	0.51	0.14	0.61
Defence†	43.33	31.53	47.33	18.38	25.67	18.84	0.22
Yellow cards †	3.33	1.51	5.50	3.08	4.33	4.23	0.49
Red cards †	0.00	0.00	0.17	0.41	0.17	0.41	0.99
Passing accuracy *	0.70	0.10	0.71	0.07	0.73	0.08	0.57
Tackle accuracy †	0.75	0.06	0.80	0.06	0.76	0.14	0.07
Minutes per match †	60.77	10.58	78.94	5.73	63.20	16.18	0.04
National less important							
Shooting accuracy †	0.44	0.11	0.43	0.17	0.44	0.07	0.85
Defence †	43.00	39.87	27.00	11.87	35.50	25.93	0.84
Yellow cards †	2.00	1.41	1.67	2.66	1.75	0.96	0.71
Red cards †	0.00	0.00	0.17	0.41	0.25	0.50	0.99
Passing accuracy *	0.64	0.09	0.65	0.09	0.68	0.06	0.04
Tackle accuracy †	0.86	0.12	0.72	0.05	0.84	0.13	0.12
Minutes per match †	55.75	18.49	51.39	12.56	55.77	24.99	0.31

* p-values of repeated measures ANOVA;

^†^ p-values of Friedman non-parametric test.

**Fig 3:**
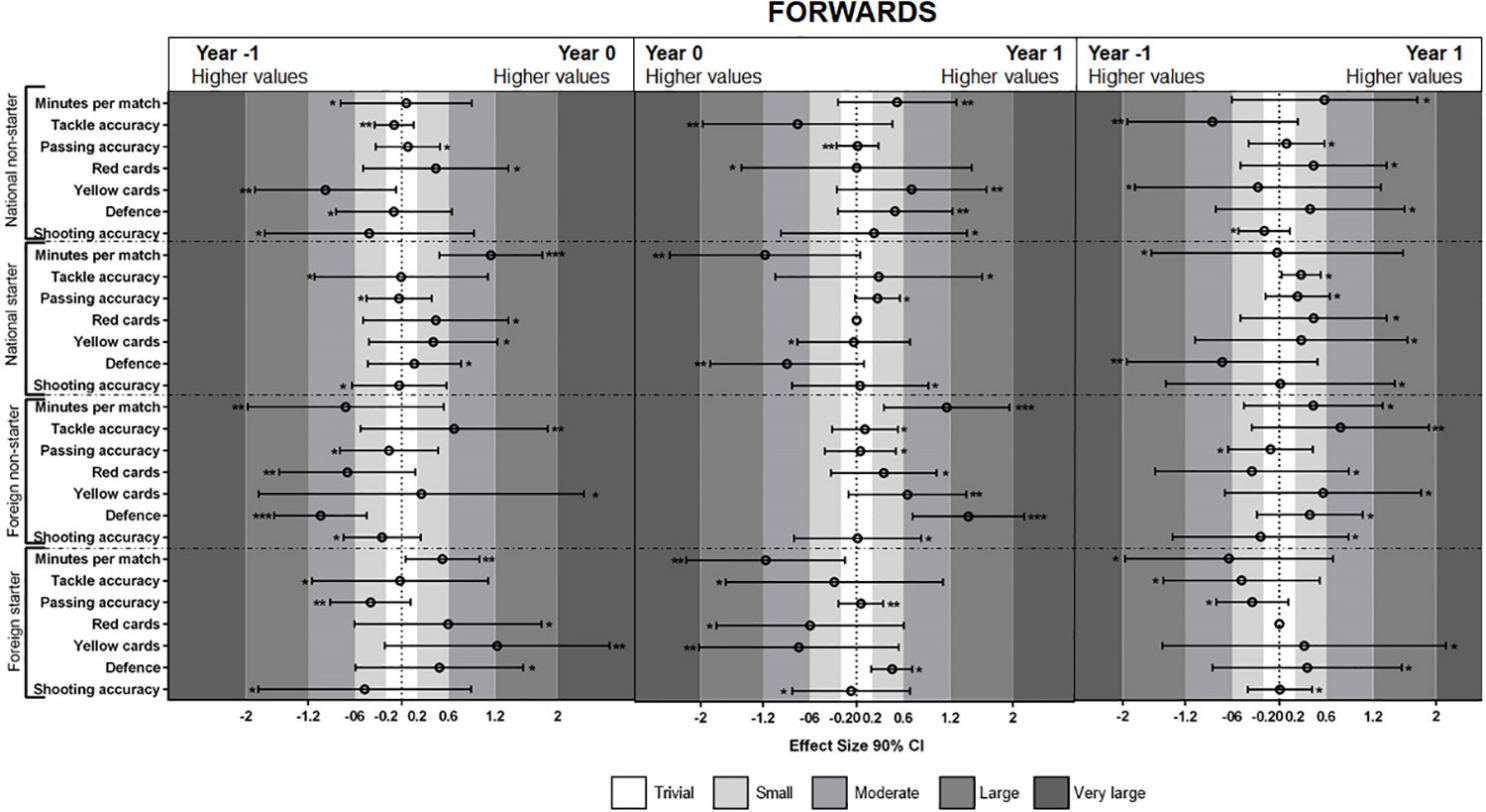
Standardized (Cohen’s d) differences between forwards players’ performances during year 0 vs. year -1, year 1 vs. year 0 and year 1 vs. year -1. Asterisks indicate the likelihood of MBI effects as follows: *possibly, **likely, ***very likely.

The MBI results for forward playing position (see Fig 3) showed that foreign less important players decrease defense performance (very likely effect) from year -1 to 0. Moreover, these players increased minutes played (very likely effect) and defense performance (most likely effect) from year 0 to year 1. Lastly, national important players increased the minutes played from year -1 to 0 (very likely effect).

## Discussion

The aim of the present study was to analyse differences in performance of individual football players depending on the previous (2-years) and the later year after signing or renegotiating a new contract, while taking player-related characteristics into account (age, role in the team as important or less important, nationality, and team’s ability). This approach enabled to gain a better understanding of the effects of signing a new contract over consecutive seasons. In contrast to the common perception among sports fans that players become lazy and expend less effort once they have signed a long-term contract [18], or conversely start to deliver better performances in order to reach a promising bargaining position in the last season before the contract expires, the present results do not provide clear support for this hypothesis. In this respect the present findings are not supportive of previous studies reporting evidence for a relationship between performance and contract duration in sports in general [21–23] and specifically in soccer [13,14, 24–27].

**Fig 4:**
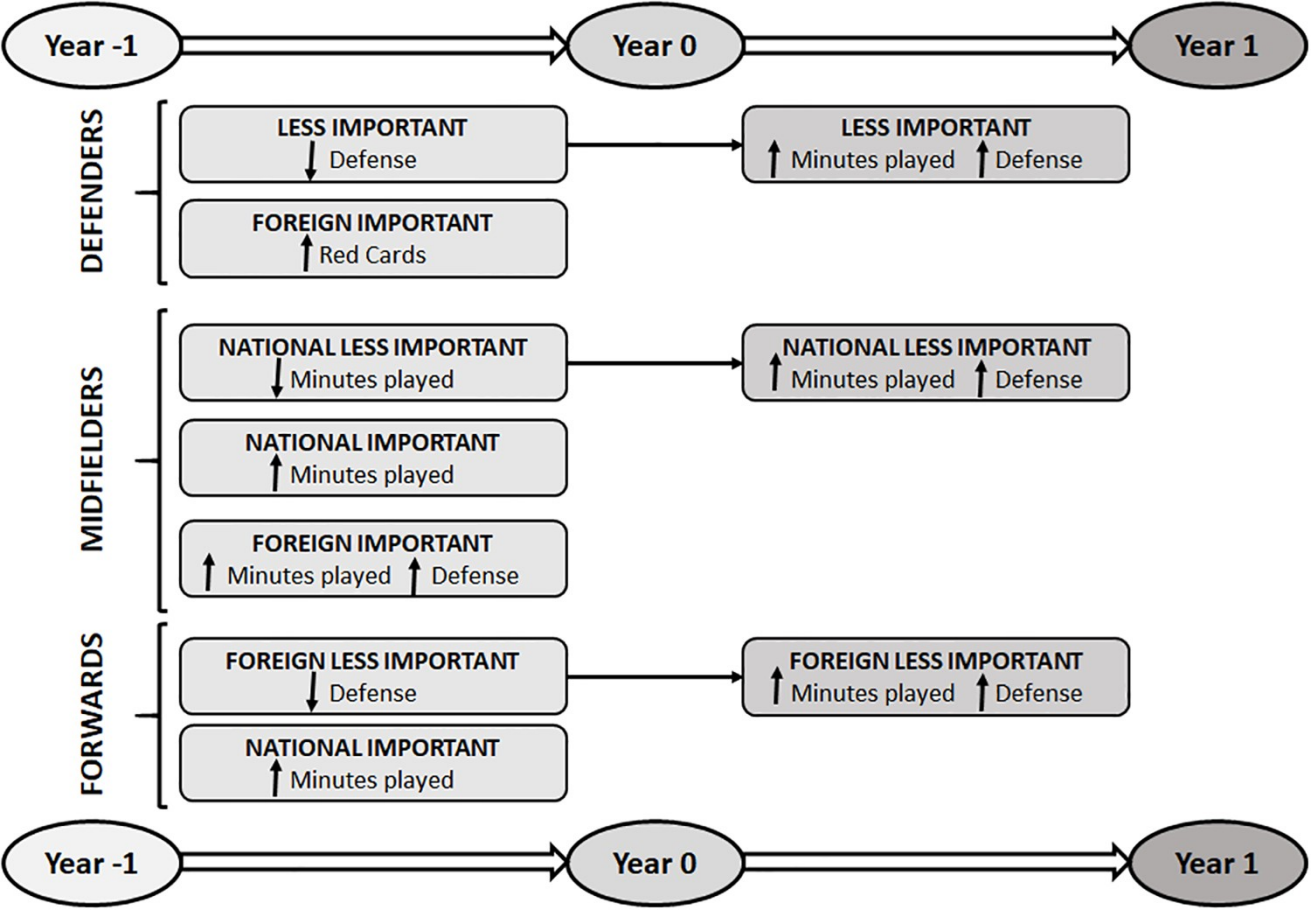
Summary of the main results (very likely and most likely effects) for each playing position and player type from year -1 to year 0 and from year 0 to year 1 (the arrows indicate the trend of player’s performance: Increased/ decreased).

Overall, our results do not demonstrate a clear association between performance and contract duration. While the minutes played and defense showed significant differences for years -1 and 1 in national less important defenders and midfielders and foreign less important forwards, with better values during the year after the new signing of a contract, no differences were found for the rest of the variables or other groups of players. Hence, the results do not support our hypothesis that performance during the previous season is better than the performances immediately after signing the new contract. On the contrary, the current results showed, for example, that performance was worse for the less important defenders (defense variable), national important midfielders (minutes played per match variable), and foreign less important forwards (defense and minutes played variables) during the previous season of signing a new contract (see Fig 4).

Previous studies suggest that the nationality of footballers may affect the relationship between performance and signing a new contract. Hence, the nationality of players can be considered as a moderator on the performance effects of signing a new contract. For example, Della Torre et al. [13] found that the individual performance during consecutive seasons is stronger for domestic players than for foreign players when the end of the contract is near. The findings of the current study do not support this evidence. There are no substantial differences between foreign and national players (see Fig 4). For example, national and foreign important midfielders increased the minutes played from year -1 to year 0. When the year after signing a contract is considered, no differences were found between national and foreign important players in any playing position. More studies are needed in order to clarify this conflicting pattern of results in the literature [13].

To the best of our knowledge, this is the first study analyzing the association between performance and contract duration depending on playing position. The current findings do not support previous studies [12–13]. According to our results, players did not perform better during the last year of their contract suggesting that maybe signing a new contract has no clear impact on the player’s motivation as it has been proposed previously. This may be due to the limit of 2-years analysis adopted in previous studies or the different performance indicators used in the current study. Future studies should scrutinize these findings.

However, managers, supporters and players should be careful when interpreting these results. Given the complexities of soccer, the match-performance trends of the players in the current study could also reflect the actions of the opposing team and teammates. Thus, individual performances could be influenced by collective strategies and tactics potentially disguising small effects that signing a new contract might have on observable individual performance indicators. Future research should possibly examine not only individual performances in isolation but also consider collective performance indicators [8–9, 28].

The present study is not without limitations. The relationships between previous and actual performances could be affected by the current salary of the players and the opportunities of signing a new contract [13]. Accordingly, the analysis of football players’ performance should be mediated by the seasonal performances of their career and their market value. These variables should be included in future works. More variables (e.g. technical, tactical and physical indicators) and countries (i.e. specific leagues with the same or different foreign recruitment policies as European competitions) should be considered to provide conclusive evidence on the relationship between performance and contract duration. Further, we did not control for: (i) the duration of the contract signed and years remaining which might result in differential effects depending on the length of the newly signed contract; (ii) if the player moves to another club or stays with the same club; or (iii) the specific playing position during each year for the players analysed. Therefore, a fruitful avenue for further research would be to conduct multifactorial analysis of signing a contract in football [12–14].

In conclusion, this paper investigated the association between performance, contract duration, and nationality of the players in elite soccer. The research does not demonstrate a clear association between performance and contract duration using datasets from the French, German, Italian, and Spanish professional leagues during the seasons 2008 to 2015. Players’ performances did not show a clear decline or improvement during the two years before signing or renegotiating a new contract. Hence, the common assumption of football spectators that players perform better when playing for a new contract and “take a break” once they signed a new contract was not identified in the present data.

## Practical applications

From a player’s recruitment or renovation perspective, the performance displayed during the past or the following seasons before or after a new contract can help managers and coaches to decide when a new contract should be signed, the duration of the contract or the salary of the player. While previous work [12–13] might be indicative of having short term contracts for players and waiting until the last year before a contract runs out to resign a player, the present results do not support this reasoning. On the contrary, players sometimes increased performance after signing a new contract.

## Supporting information

S1 Dataset IBM SPSS dataset of players’ performances obtained by OptaSports Company. (SAV)
